# The induction of stromule formation by a plant DNA-virus in epidermal leaf tissues suggests a novel intra- and intercellular macromolecular trafficking route

**DOI:** 10.3389/fpls.2012.00291

**Published:** 2012-12-27

**Authors:** Björn Krenz, Holger Jeske, Tatjana Kleinow

**Affiliations:** ^1^Plant Pathology and Plant-Microbe Biology, Cornell UniversityIthaca, NY, USA; ^2^Molecular Biology and Plant Virology, Institute of Biology, Universität StuttgartStuttgart, Germany

**Keywords:** geminivirus, movement protein, plastid, chaperone, heat shock protein

## Abstract

Stromules are dynamic thin protrusions of membrane envelope from plant cell plastids. Despite considerable progress in understanding the importance of certain cytoskeleton elements and motor proteins for stromule maintenance, their function within the cell has yet to be unraveled. Several viruses cause a remodulation of plastid structures and stromule biogenesis within their host plants. For RNA-viruses these interactions were demonstrated to be relevant to the infection process. An involvement of plastids and stromules is assumed in the DNA-virus life cycle as well, but their functional role needs to be determined. Recent findings support a participation of heat shock cognate 70 kDa protein (cpHSC70-1)-containing stromules induced by a DNA-virus infection (*Abutilon* mosaic virus, AbMV, *Geminiviridae*) in intra- and intercellular molecule exchange. The chaperone cpHSC70-1 was shown to interact with the AbMV movement protein (MP). Bimolecular fluorescence complementation confirmed the interaction of cpHSC70-1 and MP, and showed a homo-oligomerization of either protein *in planta*. The complexes were detected at the cellular margin and co-localized with plastids. In healthy plant tissues cpHSC70-1-oligomers occurred in distinct spots at chloroplasts and in small filaments extending from plastids to the cell periphery. AbMV-infection induced a cpHSC70-1-containing stromule network that exhibits elliptical dilations and transverses whole cells. Silencing of the *cpHSC70* gene revealed an impact of cpHSC70 on chloroplast stability and restricted AbMV movement, but not viral DNA accumulation. Based on these data, a model is suggested in which these stromules function in molecule exchange between plastids and other organelles and perhaps other cells. AbMV may utilize cpHSC70-1 for trafficking along plastids and stromules into a neighboring cell or from plastids into the nucleus. Experimental approaches to investigate this hypothesis are discussed.

## INTRODUCTION

In plants, transport of endogenous macromolecules such as proteins and nucleic acids over cellular boundaries occurs in a highly selective and regulated manner (Oparka, 2004; Lee and Lu, 2011; Maule et al., 2011; Niehl and Heinlein, 2011; Zavaliev et al., 2011). These controlled intra- and intercellular pathways are exploited by plant viruses for their systemic spread within their hosts; viruses can thus be used as tools to study basic endogenous transport processes within plants (Lee et al., 2003; Lucas, 2006; Benitez-Alfonso et al., 2010; Harries and Ding, 2011; Harries et al., 2011; Niehl and Heinlein, 2011; Schoelz et al., 2011; Ueki and Citovsky, 2011). There is evidence accumulating that interactions of viruses with the cytoskeleton or the endomembrane system are involved in the targeting of viral nucleoprotein complexes and transport-mediating movement proteins (MPs) to plasmodesmata. However, it is still not possible to generate a complete model of intra- and intercellular movement for any known plant virus. Considering the diverse and sometimes contrasting reports on the roles of various cellular components in viral spread, it is conceivable that viruses use fundamentally different transport mechanisms within their hosts. This seems to be the case for members within one genus, as shown, for example by research into RNA-viruses of the genus *Tobamovirus* [turnip vein-clearing virus (TVCV) and tobacco mosaic virus (TMV); Harries et al., 2009] and the genus *Potexvirus* [*Alternanthera* mosaic virus (AltMV) and potato virus X (PVX); Lim et al., 2010].

## TRANSPORT MODELS FOR THE PLANT DNA GEMINIVIRUSES

In contrast to RNA-viruses, plant-infecting DNA geminiviruses (family *Geminiviridae*) replicate within the nucleus, and systemic infection requires the crossing of two cellular barriers, the nuclear envelope via pores and the cell wall via plasmodesmata (Waigmann et al., 2004; Krichevsky et al., 2006; Lucas, 2006; Jeske, 2009). The geminiviruses have relatively small genomes (2.5–3.0 kb per single-stranded DNA circle) and with this limited coding capacity exhibit a strong dependency on host proteins to complete their life cycle. As a consequence, viral-encoded transport-mediating proteins have to interact with a variety of plant factors involved in macromolecular trafficking to overcome cellular boundaries and transfer viral DNA (vDNA) from a nucleus through the cytoplasm and via plasmodesmata into an adjacent cell and into the nucleus of that cell. The genome of bipartite geminiviruses (genus *Begomovirus*) consists of two DNA molecules: DNA A and DNA B. The two DNA B-encoded proteins, nuclear-shuttle protein (NSP) and MP, mediate the viral transport processes (Gafni and Epel, 2002; Rojas et al., 2005; Wege, 2007; Jeske, 2009) and both proteins have an impact on viral pathogenicity (Rojas et al., 2005; Zhou et al., 2007; Jeske, 2009). Previous work showed the C-terminal domain of begomoviral MPs to be important for symptom development and pathogenicity (von Arnim and Stanley, 1992; Ingham and Lazarowitz, 1993; Pascal et al., 1993; Duan et al., 1997; Hou et al., 2000; Saunders et al., 2001; Kleinow et al., 2009a). The DNA A-encoded coat protein (CP) is not essential for systemic infection of bipartite begomoviruses, suggesting that the transport complex is distinct from virions (Rojas et al., 2005; Jeske, 2009). However, CP was able to complement defective NSP mutants, and is therefore regarded as a redundant element in viral movement (Qin et al., 1998). Several studies provide evidence that NSP facilitates trafficking of vDNA into and out of the nucleus, and that MP serves as a membrane adaptor and mediates cell-to-cell transfer via plasmodesmata as well as long-distance spread through the phloem (Rojas et al., 2005; Krichevsky et al., 2006; Wege, 2007; Jeske, 2009).

Two models are currently suggested for the role of NSP and MP during cell-to-cell transport of bipartite geminiviruses: the “couple-skating” and the “relay race” models (Rojas et al., 2005; Jeske, 2009). The “couple-skating” model is based on the experimental data of the phloem-limited begomoviruses squash leaf curl virus (SLCV; Pascal et al., 1994; Sanderfoot and Lazarowitz, 1995; Sanderfoot et al., 1996), cabbage leaf curl virus (CaLCuV; Carvalho et al., 2008a,b), and *Abutilon* mosaic virus (AbMV; Zhang et al., 2001; Aberle et al., 2002; Hehnle et al., 2004; Frischmuth et al., 2007). This model suggests that MP binds the NSP/vDNA complex at the cytoplasmic side of plasma membranes or microsomal vesicles, and transfers the nucleoprotein complex into the next cell either along the plasma membrane or via the endoplasmic reticulum (ER) that spans the plasmodesmata. In contrast, the “relay race” model predicts that after NSP-mediated nuclear export the vDNA is taken over by MP, which then transports the vDNA into the adjacent cell (Noueiry et al., 1994; Rojas et al., 1998, 2005). This model is based on experimental data of the mesophyll-invading begomovirus bean dwarf mosaic virus (BDMV; Levy and Tzfira, 2011). Nevertheless, details of how both proteins co-ordinate vDNA transfer from the nucleus to the cell periphery and further throughout the plant body, are mostly unknown.

For a controlled cycle of geminiviral replication, transcription, encapsidation, and movement, NSP and MP are most likely integrated into a regulatory network consisting of other viral proteins and plant factors. Several studies have characterized a set of interacting host proteins for NSP and MP. NSPs of CaLCuV, tomato golden mosaic virus (TGMV), and tomato crinkle leaf yellows virus (TCrLYV) were found to interact with two classes of receptor-like kinases from *Arabidopsis thaliana* (Fontes et al., 2004; Mariano et al., 2004; Florentino et al., 2006). The further analysis of the NSP/kinase interactions indicated that they play a role in infectivity and symptom development. NSP counters activation of defense signaling mediated by one kinase class via phosphorylation of an immediate downstream target, the ribosomal protein L10/QM (Fontes et al., 2004; Mariano et al., 2004; Florentino et al., 2006; Carvalho et al., 2008c; Rocha et al., 2008; Santos et al., 2010). Additionally, CaLCuV NSP was found to interact with an acetyltransferase (AtNSI; McGarry et al., 2003; Carvalho and Lazarowitz, 2004; Carvalho et al., 2006) and with a small GTPase (Carvalho et al., 2008a,b). AtNSI is proposed to regulate nuclear export of vDNA by acetylating histones and CP. Carvalho et al. (2008a,b) suggest a function for the small GTPase in nuclear export processes, probably as a co-factor of NSP.

Independent of the transport model, the begomoviral MPs have to mediate multiple functions during intra- and intercellular trafficking. The identification of three phosphorylation sites in the AbMV MP, which have an impact on symptom development and/or vDNA accumulation (Kleinow et al., 2009a), indicates a regulation of diverse MP functions by yet unknown host kinases. Currently, three interacting host factors of begomoviral MPs have been identified: a histone H3 (Zhou et al., 2011), a synaptotagmin (SYTA; Lewis and Lazarowitz, 2010), and a chaperone, the heat shock cognate 70 kDa protein cpHSC70-1 (Krenz et al., 2010). Gel overlay assays, and *in vitro* and *in vivo* co-immunoprecipitation (Co-IP) experiments showed an interaction of H3 with NSP and MP of BDMV as well as with CPs of different geminiviruses (Zhou et al., 2011). In *Nicotiana tabacum* protoplasts and *N. benthamiana* leaves, transiently expressed H3 co-localized with NSP in the nucleus and the presence of MP redirected H3 to the cell periphery and plasmodesmata. A complex composed of H3, NSP, MP, and vDNA was recovered by Co-IP from *N. benthamiana* leaves transiently expressing epitope-tagged H3. The data support a model in which histone H3 is a component of a geminiviral movement-competent vDNA complex that assembles in the nucleus and is transferred to the cell periphery and plasmodesmata. SYTA localized to endosomes in *Arabidopsis* cells, and interacted with MPs of the begomoviruses CaLCuV and SLCV as well as with the unrelated MP of the RNA-virus TMV (Lewis and Lazarowitz, 2010). Transgenic *Arabidopsis* lines with either a reduced SYTA level or expressing a dominant-negative SYTA mutant exhibited a delayed systemic infection and an inhibition of cell-to-cell trafficking of the different MPs. Consequently, Lewis and Lazarowitz (2010) proposed that: (i) SYTA regulates endocytosis and (ii) distinct viral MPs transport their cargo to plasmodesmata for cell-to-cell spread via an endocytotic recycling pathway. The chaperone cpHSC70-1 of *Arabidopsis* was shown to specifically interact with the N-terminal domain of AbMV MP in a yeast two-hybrid system (Krenz et al., 2010). Bi-molecular fluorescence complementation (BiFC) analysis provided further evidence for the chaperone/MP interaction, and revealed an MP as well as a cpHSC70-1 self-interaction *in planta* (Krenz et al., 2010). MP/cpHSC70-1 complexes and MP-oligomers were observed at the cell periphery and co-localized with chloroplasts. The detection of MP-homo-oligomers at the cellular margin is in agreement with other localization studies in plant cells (Zhang et al., 2001; Kleinow et al., 2009b) and with earlier yeast two-hybrid assays that showed an MP oligomerization via the C-terminal domain (Frischmuth et al., 2004). MP-oligomer formation has also been detected at chloroplasts (Krenz et al., 2010). It is unknown whether BiFC results from MP imported into plastids or merely associated with the outer envelope of the chloroplast. No BiFC signal was seen in peri-nuclear sites as was previously found for AbMV MP transiently expressed as green fluorescent protein (GFP) fusion in plant cells (Zhang et al., 2001). Thus, MP/MP interaction may be restricted to chloroplasts and the cell periphery.

Bi-molecular fluorescence complementation showed that cpHSC70-1-oligomers were mainly associated with chloroplasts where they accumulated in distinct spots, and occurred to a lower extent in small filaments extending from plastids to the cell periphery and distributed at the periphery (Krenz et al., 2010). The localization of cpHSC70-1 was significantly influenced by AbMV-infection, accumulating in fluorescent foci on long filamental tubular structures reminiscent of plastid stromules, stroma-filled plastid tubules (Natesan et al., 2005; Hanson and Sattarzadeh, 2008). It remains uncertain whether cpHSC70-1 was maintained exclusively within the stroma or whether it was re-located to other structures upon geminiviral infection such as envelope membranes or the intermembrane space. Altogether, AbMV-infection seems to induce a prominent formation of stromules. To our knowledge the geminivirus AbMV is the only plant DNA-virus so far for which stromule biogenesis was documented. Silencing of the *cpHSC70* gene of *N. benthamiana* with the aid of an AbMV DNA A-derived gene silencing vector caused tiny white leaf sectors, which indicated an impact of cpHSC70 on chloroplast stability (Krenz et al., 2010). vDNA accumulated within these small chlorotic areas that were spatially restricted to small sectors adjacent to veins, suggesting a functional relevance of the MP/chaperone interaction for AbMV transport to symptom induction *in planta*.

## CELLULAR FUNCTIONS OF HSP70 AND HSC70 AND THEIR PUTATIVE ROLES IN VIRAL INFECTION

The expression of chaperones from the heat shock protein 70 kDa (HSP70) family is induced in response to developmental signals and various abiotic and biotic stress stimuli (Escaler et al., 2000a,b; Maule et al., 2000; Sung et al., 2001; Aparicio et al., 2005; Brizard et al., 2006; Swindell et al., 2007). Some family members exhibit a low constitutive expression level and are therefore named heat shock cognate proteins 70 kDa (HSC70s) (Sung et al., 2001; Swindell et al., 2007). The cellular functions of this chaperone family are quite diverse. They assist newly translated proteins to obtain their active conformation, misfolded or aggregated proteins to refold, assist in membrane translocation of proteins, in assembly and disassembly of macromolecular complexes and in controlling the activity of regulatory factors (Kanzaki et al., 2003; Mayer and Bukau, 2005; Weibezahn et al., 2005; Bukau et al., 2006; Noel et al., 2007; Kampinga and Craig, 2010; Mayer, 2010; Flores-Pérez and Jarvis, 2012). In addition to their intracellular functions in different subcellular compartments, HSP70s play a role in cell-to-cell transport as indicated by two non-cell-autonomous cytoplasmic HSP70s from *Cucurbita maxima* (Aoki et al., 2002) and by closterovirus-encoded homologs of HSP70s which are essential for virus transport and plasmodesmata targeting (Alzhanova et al., 2007; Avisar et al., 2008, and references therein). For HSP70s and HSC70s, substrate binding and release is regulated by a conformational change that is driven by their ATPase activity. Co-chaperones (DNAJ-like/HSP40 type proteins) assist HSP70s and HSC70s functions with their delivery and release of substrates and by enhancing ATP hydrolysis activity.

HSP70s and HSC70s transcript and protein levels are up-regulated in plants upon an infection with RNA- or DNA-viruses (Escaler et al., 2000a,b; Maule et al., 2000; Aparicio et al., 2005; Brizard et al., 2006). Accumulation of viral proteins within the cell during the infection causes stress and might thereby induce the expression of this chaperone family. Several classes of chaperones and co-chaperones including HSP70s/HSC70s and their specific co-chaperones were identified to interact with viral proteins to facilitate the regulation of viral replication, transcription, encapsidation, and intra- and intercellular movement as well as to suppress pathogen responses (Noel et al., 2007; Benitez-Alfonso et al., 2010; Nagy et al., 2011). Recently, silencing of a cytosolic HSC70-1 was found to impair infection by the monopartite geminivirus tomato yellow leaf curl Sardinia virus (TYLCSV) in *N. benthamiana* (Lozano-Duran et al., 2011). However, none of these HSP70s and HSC70s involved in viral life cycles were located in the chloroplast stroma where cpHSC70-1 was identified to interact with the MP of the geminivirus AbMV (Krenz et al., 2010). In addition to the localization of cpHSC70-1 in the chloroplast stroma and stromules, it is also seen in mitochondria and as a nuclear protein in response to cold stress (Sung et al., 2001; Peltier et al., 2002, 2006; Bae et al., 2003; Ito et al., 2006; Su and Li, 2008, 2010; Krenz et al., 2010; Latijnhouwers et al., 2010). An analysis of an *Arabidopsis* knock-out mutant of cpHSC70-1 revealed that its deficiency caused severe developmental defects (Su and Li, 2008, 2010; Latijnhouwers et al., 2010), but the functions of cpHSC70-1 and other stroma-targeted HSP70s/HSC70s are not completely understood. Recent genetic and biochemical analyses indicated that cpHSC70-1 seems to play a role in protein translocation into the plastid stroma in early developmental stages of plants (Su and Li, 2010; Flores-Pérez and Jarvis, 2012). It is well known that HSP70s/HSC70s fulfill multiple functions in chloroplasts (Flores-Pérez and Jarvis, 2012), therefore the participation of cpHSC70-1 in protein transport across membranes might not be the only function it provides. What function of cpHSC70-1 is targeted by AbMV MP? It can be speculated that the virus exploits the ATPase activity of the chaperone as a driving force to mediate transport of the geminiviral nucleoprotein complexes.

## PLASTIDS AND STROMULES IN VIRAL INFECTION

Several interactions of RNA-viruses with chloroplasts have been described which were important for the viral infection process (Reinero and Beachy, 1986; Schoelz and Zaitlin, 1989; Prod’homme et al., 2003; Jimenez et al., 2006; Torrance et al., 2006; Xiang et al., 2006; Lin et al., 2007; Lim et al., 2010). Virus–chloroplast interactions most likely facilitate viral replication or movement. The role of chloroplasts in the life cycle of plant DNA-viruses needs to be examined. In studies of cellular alterations induced by various geminiviruses in systemically infected plants, dramatic morphological changes in the ultrastructure of chloroplasts were identified, such as vesiculated entities, reduced starch and chlorophyll content, accumulation of fibrillar inclusions, virus-like particles, and vDNA within plastids (Esau, 1933; Jeske and Werz, 1978, 1980a,b; Schuchalter-Eicke and Jeske, 1983; Jeske and Schuchalter-Eicke, 1984; Jeske, 1986; Gröning et al., 1987, 1990; Rushing et al., 1987; Channarayappa et al., 1992). For AbMV it was shown that the severity of chloroplast structure remodeling was dependent on light intensity, and diurnal and seasonal conditions. Geminivirus-induced plastid alterations have thus far been interpreted to be an indirect result of the interference of viral infection with carbohydrate metabolism, mainly through a disruption in translocation via the phloem (Jeske and Werz, 1978). Nevertheless, the detection of vDNA, fibrillar inclusions, or virus-like particles within chloroplasts, suggests other functions of this interplay. Until now, only AbMV vDNA was detected in purified plastids from infected plants (Gröning et al., 1987, 1990). An artificial co-purification was excluded by thermolysin and DNase I treatment. *In situ* hybridization detected high amounts of AbMV vDNA in a low number of purified plastids, which would not be expected for a non-specific co-purification. However, so far, *in situ* hybridization of infected *Abutilon sellovianum* tissue only revealed AbMV vDNA-specific signals on plastids in rare cases (Horns and Jeske, 1991). Furthermore, the finding that an outer envelope membrane protein (Crumpled leaf) is implicated in the CaLCuV infection process also supports an involvement of chloroplasts in the geminiviral life cycle (Trejo-Saavedra et al., 2009). An interesting plastid modification detected upon AbMV-infection, was the induction of stromule biogenesis (Krenz et al., 2010).

Stromules emanate from the main body of the plastid and are confined by the outer and inner envelope membranes (Natesan et al., 2005; Hanson and Sattarzadeh, 2008, 2011). They represent a highly dynamic structure which extends, retracts, branches, bends, and sometimes releases vesicles from their tip (Gunning, 2005; Natesan et al., 2005; Hanson and Sattarzadeh, 2008). The typical diameter is <1 μm and the length is extremely variable due to their dynamic properties (Gray et al., 2001; Kwok and Hanson, 2004b; Waters et al., 2004). Stromules are distinguished from other irregular shaped plastid protrusions by their specific shape index (Holzinger et al., 2007). The movement of stromules relies on the actin cytoskeleton and the motor protein myosin XI (Kwok and Hanson, 2003; Natesan et al., 2009; Sattarzadeh et al., 2009). Differentially shaped stromules have been identified by using expression of various stroma-targeted fluorescent proteins; these include straight or branched tubules which can exhibit either randomly localized elliptical dilations that transverse the tubule length or triangular areas of expansion (Köhler et al., 2000; Hanson and Sattarzadeh, 2008; Schattat et al., 2011, 2012). For the latter type, branch formation occurs in tandem with dynamic remodeling of contiguous ER tubules (Schattat et al., 2011). Schattat and colleagues suppose that this co-alignment might originate from membrane contact points or by an exploitation of the same cytoskeletal elements for development. Single or multiple stromules may arise in all plastid types present in higher plant tissues, but their frequency varies; for example their abundance is significantly higher for achlorophylic plastids in sink tissues than for chlorophyll-containing plastids in green tissues (Köhler et al., 1997; Köhler and Hanson, 2000; Natesan et al., 2005; Hanson and Sattarzadeh, 2008; Schattat et al., 2012). Analyses of the fruit ripening in tomato showed that the formation of stromules is influenced by the plastid differentiation status and is inversely correlated with the density and size of plastids within a cell (Waters et al., 2004). Consequently, a role of stromules in sensing the number of plastids in a cell is supposed. Various abiotic and biotic stress conditions including heat (Holzinger et al., 2007), subcellular redox stress (Itoh et al., 2010), application of extracellular sucrose or glucose (Schattat and Klösgen, 2011), treatment with abscisic acid (Gray et al., 2012), colonization by an arbuscular mycorrhizal fungus (Fester et al., 2001; Hans et al., 2004; Lohse et al., 2005), and infiltration of agrobacteria (Schattat et al., 2012) were described as inducers of stromules. The formation of a dense plastid network in cells close to the main symbiotic structure during mycorrhizae formation supports a putative correlation between plastid metabolic activity and stromule biogenesis. An induction of stromules was detected as well upon viral infections (Esau, 1944; Shalla, 1964; Caplan et al., 2008; Krenz et al., 2010). RNA-virus infected sugar beets exhibit mosaic disease symptoms including mottling and yellow-green sectoring of leaves. Plastids within these yellow areas showed vesiculation and an amoeboid shape resembling stromules (Esau, 1944). Shalla (1964) described the appearance of “long appendages which extend and contracted within a few seconds” from the plastid body and vesicle formation inside chloroplasts of TMV-infected tomato leaflets. TMV-infected tobacco plants exhibited a strong induction of stromule formation (Caplan et al., 2008) just as for *N. benthamiana* plants locally infected with the DNA-virus AbMV (Krenz et al., 2010).

**FIGURE 1:**
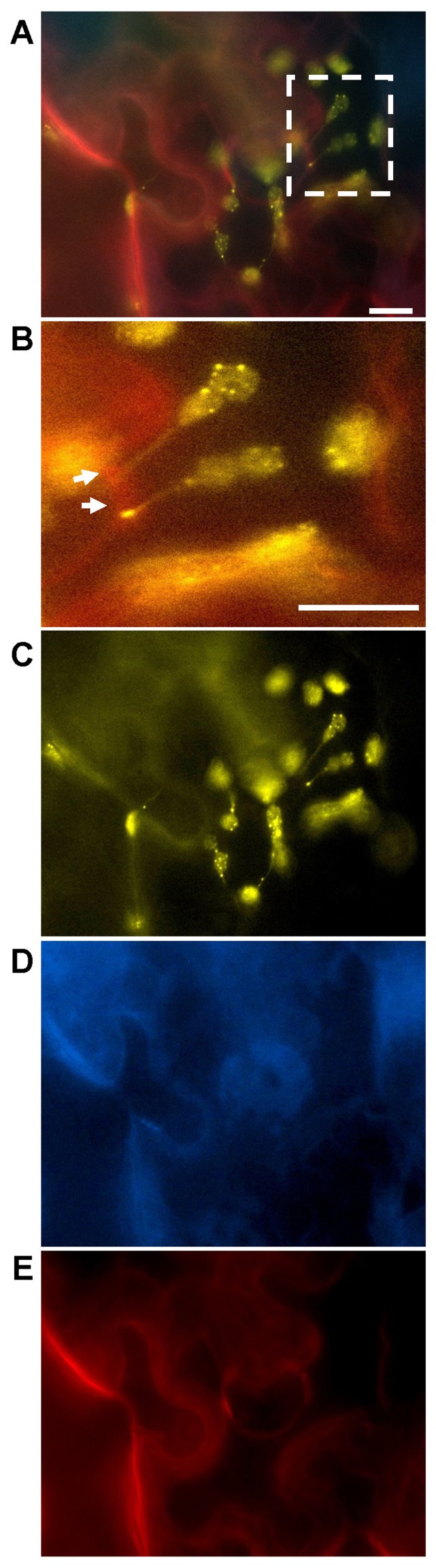
***Abutilon* mosaic virus-induced cpHSC70-1-containing stromules extending from plastids to the cell periphery.** Transient co-expression of test constructs in leaf tissues of locally AbMV-infected *N. benthamiana* and epi-fluorescence microscopy were carried out according to Krenz et al. (2010, 2011). AbMV infection was established by simultaneous agro-infiltration of infectious DNA A and DNA B clones with the fluorescent protein expression constructs. **(A)** Merged image of cells expressing NSP:cyan fluorescent protein (CFP), the two split yellow fluorescent protein (YFP)/BiFC constructs of cpHSC70-1 and the plasmodesmata marker PDCB1:mCherry (callose binding protein 1, Simpson et al., 2009) for 4 days post agro-infiltration (dpai). The square in **(A)** highlights cpHSC70-1-oligomers at chloroplasts and stromules (yellow, arrows) anchoring at the cell periphery (red: PDCB1:mCherry), and is magnified in **(B)**. The separate fluorescence signals superimposed in **(A)** are shown in **(C)** YFP, **(D)** CFP, and **(E)** mCherry. Note: The plasmodesmata marker PDCB1:mCherry lost its extracellular localization at the neck region of plasmodesmata upon AbMV-infection (compare **Figure 3**) and is probably distributed throughout the apoplast. NSP:CFP is redirected from the nucleus to the cell periphery, probably the plasma membrane, by presence of MP or AbMV-infection (Zhang et al., 2001; Frischmuth et al., 2007). Bar: 10 μm.

**FIGURE 2:**
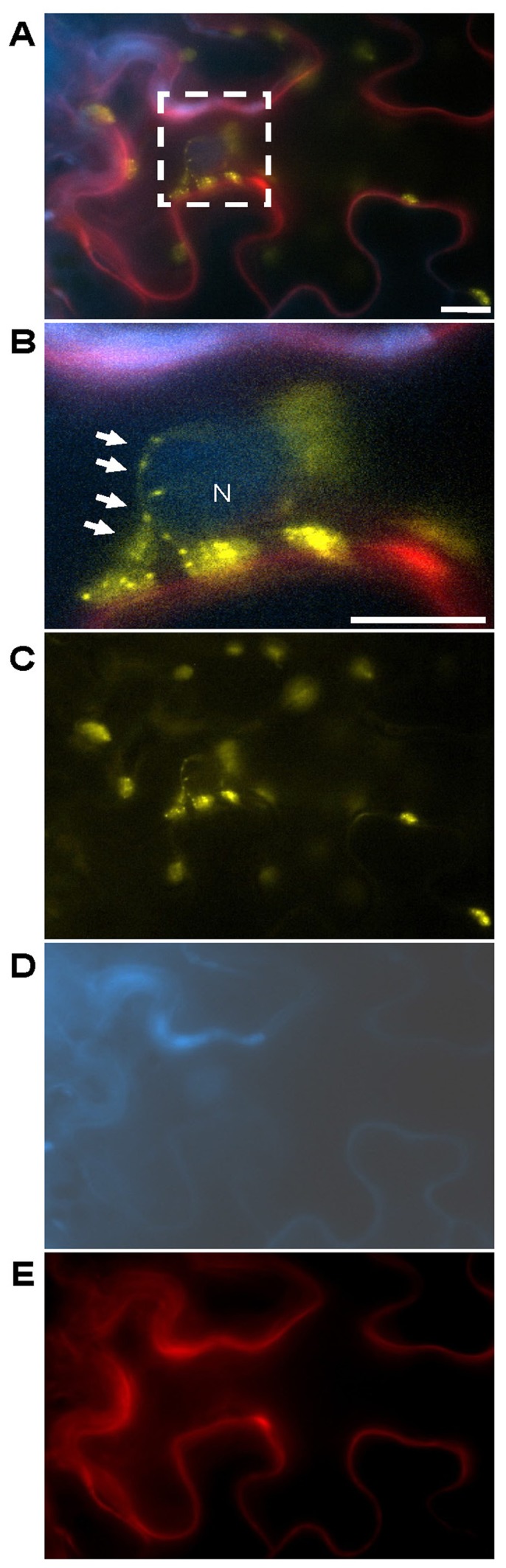
***Abutilon* mosaic virus-induced cpHSC70-1-containing stromules grabbing a nucleus**. The experimental set-up is the same as stated in **Figure 1**. **(A)** Merged image of cells expressing the three test proteins for 4 dpai and **(B)** magnification of the square in **(A)** which marks cpHSC70-1-oligomers at chloroplasts and stromules (yellow, arrows) which closely associate to a nucleus (blue: NSP:CFP) near to the cell periphery (blue: NSP:CFP, likely plasma membrane and red: PDCB1:mCherry, apoplast), magnified in **(B)**. The separate emissions merged in **(A)** are shown in **(C)** YFP, **(D)** CFP, and **(E)** mCherry. N, nucleus; bar: 10 μm.

Although several inducers of stromules have been identified, their

functional role remains to be determined. They were proposed to

participate in plastid motility and in facilitating transport of various molecules, e.g., proteins, metabolites and signaling components, into and out of a plastid, among plastids and even between plastids and other organelles (Köhler et al., 1997, 2000; Kwok and Hanson, 2004a; Natesan et al., 2005; Hanson and Sattarzadeh, 2008, 2011). Chlorophyll, thylakoid membranes, plastid DNA, and ribosomes have not been detected within stromules (Hanson and Sattarzadeh, 2008, 2011; Newell et al., 2012). Nevertheless, the possibility of a rare movement of plastid DNA and ribosomes or the transfer of much smaller DNA molecules, e.g., plastid transformation vectors, via stromules cannot be completely excluded. Exchange of stroma-targeted GFP between two plastids interconnected by stromules was observed using fluorescence recovery after photobleaching (FRAP) experiments in tobacco and *Arabidopsis* (Köhler et al., 1997; Tirlapur et al., 1999). Moreover, two-photon excitation fluorescence correlation spectroscopy revealed two different transport modes through stromules in tobacco suspension cells (Köhler et al., 2000). A simple diffusion of single stroma-targeted GFP molecules was observed in addition to an active ATP-dependent batch movement of GFP “packets.” Köhler and colleagues supposed that these GFP bodies represent an accumulation of GFP in small vesicles. Stromules may carry several of these GFP “packets” leading to a beaded appearance (Köhler and Hanson, 2000; Pyke and Howells, 2002; Hanson and Sattarzadeh, 2008, 2011). The stromules induced by AbMV-infection and containing the MP-interacting chaperone cpHSC70-1 exhibited a related appearance like pearls on a string (Krenz et al., 2010; **Figures 1 and 2**). It is hypothesized that cpHSC70-1 is present in the same type of “packet” structure as GFP in the preceding experiments, probably associated with vesicles and actively transported.

The data obtained by Kwok and Hanson (2004a) suggested that stromules may serve as pathways between nuclei and more distant regions of the cell and possibly even other cells. They observed that clusters of plastids around nuclei are capable of extending stromules both outward, to the cell membrane, as well as inward, through nuclear grooves. Close contact between plastids and the nuclei and the plasma membrane of plant cells suggests that physical interactions may enhance functional interactions between these organelles. Furthermore, Kwok and Hanson (2004a) found that stromules from two adjacent cells appeared to meet at either side of an adjoining cell wall. Consequently, the stromule’s structure seems to be suitable for the exchange of molecules between plastids and other organelles or the trafficking of plastidal proteins and metabolites to diverse regions of the plant cell. Remarkably, the cpHSC70-1-containing stromules detected upon an AbMV-infection arose not only on plastids clustered around and in close association with the nucleus, but also appeared to interconnect plastids and extend from plastids outward to the cell periphery (Krenz et al., 2010; **Figures 1 and 2**).

By contrast, in non-infected tissues only short cpHSC70-1-containing filaments were found which extended solely from cortex positioned plastids to the cell periphery. However, molecular transfer from the plastids to the nucleus or vice versa with the aid of stromules remains to be confirmed. That a retrograde protein exchange between plastids and the nucleus can occur was demonstrated recently. Plastid-encoded HA-tagged Whirly1 protein was translocated to the nucleus in transplastomic tobacco plants, where it stimulated pathogen-related gene expression (Isemer et al., 2012). The chloroplast-localized NRIP1 (N receptor-interacting protein 1) was redirected to the cytoplasm and to the nucleus in presence of the p50 effector, a 50 kDa helicase domain encoded by TMV (Caplan et al., 2008). Upon this recruitment to the nucleus and the cytoplasm NRIP1 binds to the N innate immunity receptor to initiate effector recognition and pathogen defense mechanisms. TMV-infection causes a strong increase in stromule formation, and a localization of fluorescent protein-tagged NRIP1 within stromules was observed (Caplan et al., 2008). Thus, the authors speculated about an involvement of stromules in the nuclear re-localization of NRIP1.

Schattat et al. (2012) do not support a function of stromules in trafficking of macromolecules between plastids. In this thoroughly performed work, interconnectivity of independent plastids was tested with the aid of a photoconvertible stroma-targeted fluorescent protein. Despite the strong microscopic impression of interplastid connectivity via stromules, an exchange of the stroma marker protein could not be visualized by high quality confocal imaging. Various plant materials (e.g., *N. benthamiana* and *Arabidopsis*) were comprehensively analyzed for plastid morphology and marker protein transfer. Although the differently colored plastids and stromules were in very close proximity, the labeled organelles remained separate as indicated by the absence of color mixing. That the method applied in these studies is suitable to detect an exchange of material between organelles upon a fusion was confirmed by analogous experiments using a mitochondria-targeted version of the fluorescent protein. In contrast to our observations from geminivirus-infected plants, the results of Schattat et al. (2012) were obtained working with uninfected plants. Whether these conflicting results are caused by the different experimental set-up, plastid types, and plant material used, or whether indeed a macromolecular trafficking of stroma-proteins through interconnecting stromules is not feasible under any conditions, needs to be elucidated by further experimentation.

**FIGURE 3:**
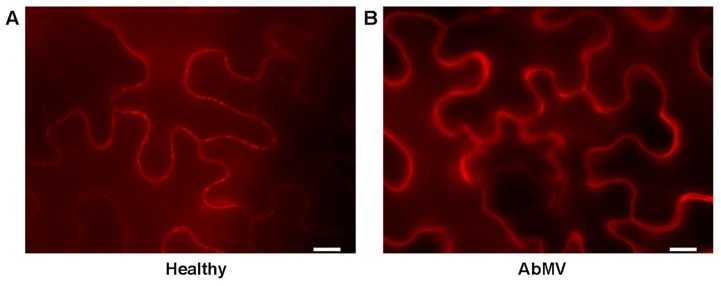
**Influence of AbMV-infection on localization of the plasmodesmata marker PDCB1.** PDCB1:mCherry (Simpson et al., 2009) was expressed in epidermal tissues of *N. benthamiana* plants either **(A)** healthy or **(B)** locally infected with AbMV for 4 days. The experiment was performed as described in **Figure 1**. **(A)** Punctate mCherry signals indicate the targeting of PDCB1 to the apoplastic part of the neck region from plasmodesmata according to Simpson et al. (2009). **(B)** Upon AbMV-infection these plasmodesmata-specific signals disappeared and PDCB1 emerged homogenously in the apoplast. These results showed a virus-induced alteration of the subcellular localization of PDCB1, probably by modifying callose deposition at plasmodesmata. Bar: 10 μm.

In addition to the induction of stromule biogenesis, AbMV-infection influences the localization of a plasmodesmata-associated protein. The plasmodesmata callose binding protein 1 (PDCB1) fused to mCherry was investigated as a marker for plasmodesmata by Simpson et al. (2009). PDCB1 is a glycosylphosphatidylinositol (GPI)-linked protein that exhibits callose binding activity and localizes to the neck region of plasmodesmata in the apoplast. Here it possibly acts as a structural anchor between the plasma membrane component spanning the plasmodesmata and the cell wall. The available data support a function for PDCB1 in plasmodesmata flux control by influencing the callose deposition in the cell wall and as a consequence the aperture of plasmodesmata. Due to its extracellular localization PDCB1 was not expected to interfere with viral proteins like AbMV MP, which is likely to accumulate in the central symplastic cavity region of complex plasmodesmata (Kleinow et al., 2009b; Lee and Lu, 2011). The fluorescence microscopic analyses showed punctate structures in the cell periphery after transient expression of PDCB1:mCherry in epidermal leaf tissues, which are in agreement with the expected plasmodesmata localization (**Figure 3**).

Surprisingly, upon AbMV-infection PDCB1:mCherry signals were still distributed at the cell periphery, most likely the cell wall (**Figures 1–3**), but no punctate structures were detected anymore. Thus, the protein seems to have lost the plasmodesmata localization. We hypothesize this as an AbMV-induced remodeling of the plasmodesmata aperture by callose depletion in the neck region.

## AbMV MOVEMENT ALONG STROMULES WITH THE HELP OF A CHAPERONE

A cellular function in plant endogenous macromolecular trafficking is suspected for stromules and chaperones like HSC70s and in addition an involvement for chaperones in viral movement. However, their combined participation in these processes has thus far not been examined. The accumulated data for the geminivirus AbMV indicate that for both factors there is a joint involvement in the viral life cycle, very likely the movement process (Krenz et al., 2010). In the studies of Krenz et al. (2010) stromules were visualized on chlorophyll-containing plastids by BiFC experiments using the chaperone cpHSC70-1. Here, two different types of cpHSC70-1-containing stromules were monitored. Only short stromules extending from plastids to the cell periphery have been found in healthy epidermal tissues, whereas upon AbMV-infection long stromules forming a network between plastids, nucleus, and the cell periphery were detected. For the latter ones, the BiFC signals of cpHSC70-oligomers highlighted mainly stromule structures with elliptical dilations giving them a beaded appearance. This significant difference created by the geminivirus infection might indicate additional functions of cpHSC70 in association with the two stromule types observed. The cpHSC70-containing stromules may function in macromolecule transfer, perhaps just under certain cellular conditions, e.g., a virus infection. This transport may happen intracellulary among plastids and between plastids and other organelles, or even intercellularly through plasmodesmata.

A prerequisite for the traveling of stromal proteins via stromules from an individual plastid to another plastid or organelle (e.g., nucleus) might be the fusion of the outer and inner envelope membrane with the target membrane. AbMV-infection might create a cellular environment that allows such a fusion event of stromules and the consequent transposition of quantities of stromal components. Alternatively, a transfer process might be initiated, which does not comprise a fusion of the inner envelope membrane or the envelope at all. This might consist of transport along the cytoplasmic leaflet of the outer envelope membrane, through the intermembrane space after fusion of the outer envelope with the target membrane or via envelope-coated vesicles released from stromules (Gunning, 2005; Krause and Krupinska, 2009). Irrespective of the underlying mechanism, a movement of a geminiviral nucleoprotein complex in association with interacting plastid stromules and cpHSC70, even with a low efficiency, might be sufficient for intra- and intercellular viral spread. A transport event in close association with membranes or vesicles would be consistent with the geminiviral MP being a membrane-associated protein. AbMV MP was localized to the protoplasmic face of plasma membranes and vesicles, where its C- and N-terminal domains most likely protrude into the cytoplasm (Aberle et al., 2002; Frischmuth et al., 2004, 2007). The central part of MP probably forms an amphipathic helix structure which inserts into one leaflet of the target membrane (Zhang et al., 2002). It has been observed that insertion of amphipathic helices into a monolayer induces bending and generates local curvature (Kozlov et al., 2010; McMahon et al., 2010). Such protein-mediated membrane stresses were found to trigger fusion, fission, and budding events of membranes. Presumably, AbMV MP may be capable of inducing and/or enhancing such membrane remodeling. However, it cannot be excluded that the close association of AbMV MP with plastid structures represents a targeting of the cytoskeletal elements, to which chloroplasts and stromules are usually attached, for cellular transit of viral nucleoprotein complexes. The result of cpHSC70 silencing in *N. benthamiana* revealed that the chaperone/MP interaction is not essential for the systemic spread of AbMV (Krenz et al., 2010), suggesting it plays a role via an alternative path.

**FIGURE 4:**
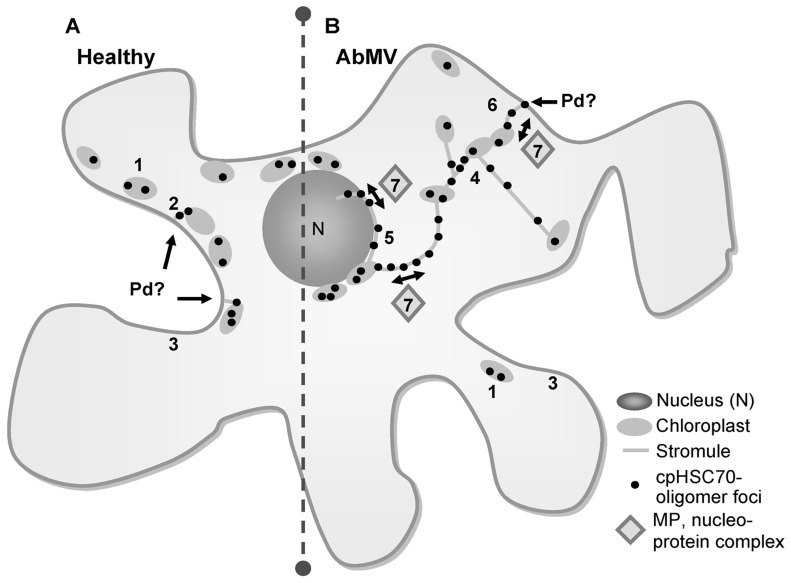
**Hypothetical model of AbMV intra- and intercellular trafficking via a plastid network.** **(A)** In healthy plant cells oligomers of the chaperone cpHSC70 locate mainly in small spots at chloroplasts (1), to a lesser amount in small filaments extending from cortical chloroplasts toward the cell periphery (2) and distributed at the cellular margin (3). **(B)** In AbMV-infected cells homo-oligomers of cpHSC70-1 were found similar to non-infected cells at chloroplasts (1) and to a low extent at the cellular margin (3). However, AbMV-infection establishes the formation of a stromule network interconnecting different chloroplasts (4), but also extending from plastids inward to the nucleus, where they closely attach (5) and outward to the cell periphery/cell wall, assumedly to plasmodesmata (Pd) that transverse the cell wall (6). These stromules exhibit structures where cpHSC70-oligomers appeared mainly in elliptical dilations giving them a “pearls on a string”-appearance. The stromule network might function in intra- and intercellular trafficking of viral nucleoprotein complexes by the interaction of AbMV MP with the chaperone cpHSC70 within stromules (7). The potential underlying transport mechanism is yet unknown, but probably involves membrane fusions or a vesicle formation.

In summary, a model (**Figure 4**) can be proposed in which MP/cpHSC70 interaction and stromule induction facilitate intra- and intercellular macromolecular trafficking along plastids and stromules into the neighboring cell or in the other direction from plastids into the nucleus. Whether this represents an accidental event or is of major significance for AbMV propagation and/or symptom development remains to be investigated.

So far geminivirus replication and virion assembly were only detected within the nucleus of infected cells (Rojas et al., 2005; Jeske, 2007, 2009). Thus, it seems to be very unlikely that the observed interaction of AbMV with plastids and stromules plays a role in replication or virion assembly processes. Nevertheless, AbMV-induced stromule formation and/or cpHSC70-1 interaction might be also related to other cellular processes. Plastids were involved in the biosynthesis pathways of many essential compounds (e.g., carbohydrates, fatty acids, purines) and stromules might be important in facilitating metabolic exchanges within the cells (Fester et al., 2001; Hans et al., 2004; Kwok and Hanson, 2004b; Waters et al., 2004; Lohse et al., 2005; Schattat and Klösgen, 2011). Plant cells respond to various abiotic and biotic stress stimuli, causing disturbance in the cellular energy status, by complex changes, which include the carbohydrate metabolism and therefore also plastid activity. It is known that stromules occur more frequently and are longer in plant cells with disturbed metabolism (Hanson and Sattarzadeh, 2008), e.g., cells cultured in liquid medium, callus or suspension culture that shed chlorophyll in their chloroplast. That stromule emergence is triggered by an increased plastid metabolic capacity resulting from biotic stress, is supported by the findings of their strong induction upon symbiotic interaction of root cells with mycorrhiza (Fester et al., 2001; Hans et al., 2004; Lohse et al., 2005). Interestingly, geminiviral proteins other than MP have an impact on a regulatory key component of the stress and glucose signal transduction, the sucrose non-fermenting 1-related (SnRK1) protein kinase (Kong and Hanley-Bowdoin, 2002; Hao et al., 2003; Shen and Hanley-Bowdoin, 2006). Plastids and HSP70s were previously concluded to be involved in the onset of a virus-pathogen response (Noel et al., 2007; Caplan et al., 2008; Nagy et al., 2011); therefore, the interaction of AbMV with the plastidal cpHSC70-1 might depict a counteraction of an antiviral defense mechanism.

Recently, a mutant screen identified a plastid- and a mitochondria-localized RNA helicase to be necessary for plastid development, embryogenesis and unexpectedly for cell-to-cell trafficking (Burch-Smith et al., 2011; Burch-Smith and Zambryski, 2012). For both knock-out plants profound changes in the transcriptome were observed, e.g., a dramatic down-regulation of nucleus-encoded plastid-related genes. Although the proteins are located exclusively in plastids and mitochondria, respectively, loss of one of the RNA helicase functions causes formation of twinned and branched plasmodesmata in *Arabidopsis*. Thus, disruption of the plastid function seems to induce an increased molecule exchange, possibly metabolites or signaling factors, among neighboring cells. The data supports a pathway linking intra- to intercellular communication and therefore a signaling from organelles to the nucleus and plasmodesmata. It can be speculated that viral interactions with plastids also targets this novel regulatory pathway to enhance plasmodesmata trafficking.

## CONCLUSION AND FUTURE PERSPECTIVES

The AbMV data on movement is consistent with cell-to-cell transport according to the “couple-skating” model. However, the results of Krenz et al. (2010) may demand to widen this concept of geminiviral cellular transfer for AbMV: it may opportunistically hijack different pathways for intracellular transport and plasmodesmata targeting including an alternative route via chloroplasts and stromules with the aid of a plastidal chaperone. It is clear, in any case, that further research is needed to elucidate this hypothesis.

A functional characterization of AbMV MP and the host factor cpHSC70-1 in the assumed transport processes could start with comprehensive analyses of their localization in subcellular compartments and membrane structures. The following points should be considered: (i) To resolve the geometric relation of the different cellular structures high resolution confocal imaging and time lapse imaging is required. (ii) Test protein expression should be done in combination with a set of fluorescent marker proteins, probably photoconvertible, for different compartments to monitor the subcellular distribution, temporal activities, and macromolecular trafficking events. A cytoplasmic marker would be needed to investigate if AbMV-induced stromules actively associate with the nucleus or are just pressed by other organelles, like a large vacuole, toward the nucleus. (iii) Careful controls should be included to account for stresses (including agro-infiltration) that are known to influence stromule formation. (iv) To minimize the influence of agro-infiltration, transgenic plants expressing required fluorescent test proteins and alternate methods to introduce AbMV (biolistic bombardment of vDNA) should be applied. To investigate the precise molecular function of cpHSC70-1 on AbMV infection and presumably macromolecular trafficking, the impact of the following scenarios on AbMV spread could be investigated: (i) transgenic plants overexpressing wild-type cpHSC70-1 or a non-MP-interacting, dominant-negative cpHSC70-1 variant and (ii) infection with AbMV DNA encoding a non-cpHSC70-1-interacting MP mutant. Finally, the relevance of the findings should be tested for additional geminiviruses other than AbMV. This knowledge gained will contribute significantly to the elucidation of the geminiviral intra- and intercellular movement process.

## Conflict of Interest Statement

The authors declare that the research was conducted in the absence of any commercial or financial relationships that could be construed as a potential conflict of interest.
